# A decade since sulfonamide-based anti-malarial medicines were limited for intermittent preventive treatment of malaria among pregnant women in Tanzania

**DOI:** 10.1186/s12936-018-2565-1

**Published:** 2018-11-06

**Authors:** Alphonce I. Marealle, Dennis P. Mbwambo, Wigilya P. Mikomangwa, Manase Kilonzi, Hamu J. Mlyuka, Ritah F. Mutagonda

**Affiliations:** 1 Department of Clinical Pharmacy & Pharmacology, School of Pharmacy, Muhimbili University of Health & Allied Sciences, Dar es Salaam, Tanzania; 2 Muhimbili University of Health and Allied Sciences, Dar es Salaam, Tanzania

**Keywords:** Sulfonamide-based antimalarials, Availability, Medicine dispensers, Knowledge, Practice

## Abstract

**Background:**

Despite the development of resistance to *Plasmodium falciparum* malaria, sulfadoxine–pyrimethamine is still effective for intermittent preventive treatment of malaria in pregnancy (IPTp). In Tanzania, more than 10 years have passed since sulfadoxine–pyrimethamine and sulfamethopyrazine–pyrimethamine (SPs) were reserved for IPTp only. However, the retail pharmaceutical outlet dispensers’ knowledge and their compliance with the policies have not been recently explored. Therefore, this study was designed to investigate dispensers’ knowledge about these medications together with their actual dispensing practices, a decade since they were limited for IPTp use only.

**Methods:**

This descriptive cross-sectional study was conducted between February and July 2017 in all municipalities of Dar-es-Salaam city. Data were collected by direct interviews using a structured questionnaire to assess knowledge and a simulated client approach was used to assess the actual practice of medicine dispensers. Data analysis was done by using SPSS version 20 and Chi square test was used to test significant differences in proportions between different categorical variables. A p-value of less than 0.05 was considered to be statistically significant.

**Results:**

A random sample of 422 medicine dispensers participated in this study whereby 185 (43.8%) were from community pharmacies and 237 (56.2%) from accredited drug dispensing outlets. The study revealed that SPs were available in 76% of the community pharmaceutical outlets in Dar es Salaam. In general majority of the dispensers (64%) had moderate to high knowledge about SPs and their indication. About 80% of the dispensers were aware that SP is reserved for IPTp. However, irrespective of the level of knowledge, almost all dispensers (92%) were willing to dispense the medicines for the purpose of treating malaria, contrary to the current Tanzania malaria treatment guideline.

**Conclusion:**

Majority of the medicine dispensers in the community pharmaceutical outlets were knowledgeable about SPs and their indications. Disappointingly, almost all dispensers irrespective of their levels of knowledge were willing to dispense SPs for treatment of malaria contrary to the available treatment guidelines.

## Background

Malaria is still a problem in sub-Saharan Africa. Tanzania has the third largest population at risk of malaria whereby over 90% of its population lives in areas where malaria transmission is high [1].

Each year, approximately 10–12 million people contract malaria in Tanzania and 80,000 die from the disease, mostly children [2]. Anti-malarial medicines like any other anti-infectious agents have been facing resistance challenges which necessitated numerous changing of regimens. In line with World Health Organization (WHO) recommendations, Tanzania has modified its malaria treatment guidelines several times, for example in 2001 changed from chloroquine to sulfonamides containing anti-malarial medicines and in 2006 was changed to artemisinin-based combinations [3–5].

In areas of moderate to high malaria transmission, by the time an individual reaches adulthood, he/she has acquired partial immunity that can protect him/her against severe disease, but during pregnancy malaria usually tends to be asymptomatic [6–8]. Parasites may be present in the placenta and contribute to poor birth outcomes even in the absence of documented peripheral parasitaemia. In these settings, consequences of *Plasmodium falciparum* infection in pregnancy are most pronounced for women especially in their first pregnancy [9].

Even with the availability of effective medications and modern diagnostic techniques for malaria, malaria infection during pregnancy is still a significant public health problem with extensive risks for the mother, fetus, and the newborn. Malaria-associated maternal illness and low birth weight are mostly the result of *P. falciparum* infection and occur predominantly in malaria-endemic regions including Tanzania [10, 11]. In trying to tackle this problem, the WHO recommends several strategies for prevention and treatment of malaria during pregnancy including; the use of long-lasting insecticidal nets (LLINs), intermittent preventive treatment (IPTp) using sulfadoxine–pyrimethamine (SP), prompt diagnosis and effective treatment of malaria infections [12]. The regimen for IPTp-SP administration have changed over time due to wide spread of *P. falciparum* resistance to SP; initially at least two doses of IPTp-SP were given during routine antenatal care and, in 2013, the WHO issued a new IPTp guideline in which pregnant women are to receive three or more doses of SP through direct observed therapy (DOT) [12, 13].

Despite the reported failure in the treatment of malaria, SP is still effective in preventing placental malaria thereby improving maternal and fetal birth outcomes. It has been reported to reduce maternal malaria episodes, maternal and fetal anaemia, placental parasitaemia, low birth weight, and neonatal mortality [14–18]. However IPTp-SP effectiveness is threatened in areas where prevalence of the sextuple Pfdhps-A581G mutation exceeds 10%, which is the case in northern Tanzania, western Kenya and southern Uganda [19–21]. SPs are relatively cheaper than artemisinin-based combinations, and they are taken as a single dose, explaining why it is very likely for people to prefer them for treatment of malaria [22]. Misuse of SPs might encourage emergence of other new resistant strains and increase the burden of total management cost incurred due to unnecessary hospitalization as a result of disease progression to severe condition thus increase morbidity and mortality rates.

Several efforts are also been made in searching for antimalarial medicines which could substitute IPTp-SP. Dihydroartemisinine–piperaquine (DHP) has been found in Kenya to be promising alternative medicine to replace SP for IPTPp, but more studies are still needed to investigate its efficacy, safety, operational feasibility, and cost-effectiveness [23]. A larger, multicentre, randomized, non-inferiority trial in four West African countries compared the effectiveness of IPTp-SP against intermittent screening and treatment with artemether–lumefantrine (ISTp-ALu) in the control of malaria in pregnancy in areas with low prevalence of SP resistance in which it was suggested that ISTp-ALu could be used as alternative to IPTp-SP in areas with high *P. falciparum* resistance to SP or in situations where SP is contraindicated [24]. However, results from a clinical trial indicate that, IST-DHP was not a suitable alternative strategy to IPTp-SP, even in areas with high SP resistance like Kenya [23]. The combination of azithromycin and chloroquine has also been reported to be effective in the treatment of uncomplicated malaria and promising as a substitute regimen for SP in IPTp [25, 26]. Despite the efforts to search for an alternative strategy and/or medicine to substitute SP, SP is still the only medicine for IPTp in Tanzania mainland.

If SPs are dispensed and used haphazardly in the community they could be rendered ineffective for IPTp. Studies conducted in Kenya and Ghana have revealed existence of medicine retailers who have adequate knowledge regarding malaria treatment guidelines but yet do not adhere to them [27, 28]. In Tanzania, although more than a decade has elapsed since the ministry responsible for health limited SPs for IPTp it is still not clear whether the retail pharmaceutical outlet dispensers are well knowledgeable about these medications, their indications and whether they practice according to the available guideline. It is also not clear whether retail pharmaceutical outlets still stock these medications since they are supposed to be provided as DOT at the antenatal clinics. Therefore, the aim of this study was to investigate the availability of SPs at the retail pharmaceutical outlets, to assess knowledge of pharmaceutical outlet dispensers on indication of SPs, and to determine their actual practice.

## Methods

### Study design and study area

This descriptive cross-section study was conducted between February and July 2017 in all municipalities of Dar-es-Salaam which include Kinondoni, Ilala, Ubungo, Kigamboni and Temeke.

### Study population

Medicine dispensers working in retail community pharmacies and accredited drug dispensing outlets (ADDO) in Dar-es-Salaam were involved in this study.

### Sample size

Four hundred and twenty-two (422) community medicine outlets were randomly selected for the purpose of this study. There were 587 retail pharmacies and 750 ADDO shops registered in Dar-es-Salaam. To have a representative sample of study facilities the ratio of pharmacy to ADDOs to be visited was determined. Based on the ratio obtained 185 retail pharmacies and 237ADDO shops were selected randomly to make total of 422 premises. Ballot technique was applied to obtain the pharmacies and ADDO shops to be visited. During the assessment of dispensers’ practice using a simulated client, only one dispenser who attended the simulated client was assessed per sampled premise. Additionally, for each premise only one dispenser was recruited to participate in the direct interview.

### Data collection

Structured questionnaires and checklists were used for data collection through direct interview and simulated client approach, respectively. Two visits were made per every sampled pharmaceutical outlet premise. The first visit was for simulated client approach and data were collected in the simulated client checklist and the second round on a different day was for the direct interview using structured questionnaire.

### Data analysis

Data collected were analysed quantitatively using a statistical package for social science (SPSS) program version 20.0. Chi square test was used for testing significance between the study variables whereby p-value of less than 0.05 was considered statistically significant.

### Assessment of knowledge

Data on dispensers’ knowledge were obtained through structured interviews using a pre-tested questionnaire which was developed based on previous knowledge assessment studies which are closely related to this study [27, 29]. The questionnaire helped in gathering dispensers: knowledge on the current recommended indication of SP in Tanzania, knowledge on the recommended first-line treatment of uncomplicated malaria in Tanzania, knowledge on the SP dose and dosing frequency to assure protection of the pregnant women against malaria, knowledge on the contraindications of SPs and knowledge on best piece of advice to the patient dispensed with SPs like the need to take the medications with plenty of water to prevent kidney problems. Eight multiple choice questions were used where each question carried one mark and eventually total score were converted into 100% then Blooms’ cut off point was used in knowledge scoring as follows; those who scored from (0–59)%, (60–79)% and (80–100)% were regarded as having poor, moderate and high knowledge, respectively.

### Assessment of actual practice

Simulated client technique was used during assessment of actual practice whereby the researcher imitated as if he was suffering from malaria and needed sulfonamide-based anti-malarial for his treatment. After requesting for the medication the researcher observed if the dispenser was willing to dispense such anti-malarial medicines for malaria treatment. Whoever dispensed or was willing to dispense such medicines for malaria treatment was considered as practicing contrarily to the current malaria treatment guideline.

### Availability of SPs

Survey for availability of SPs at all sampled facilities was conducted during the interview visit. The data collector asked for whether SPs were available or not at all the 422 facilities visited.

## Results

### Demographic characteristics

A total of 422 dispensers from both community pharmacies (44.8%) and ADDO shops (56.2%) were enrolled in this study. Majority of the study participants were ADDO trained personnel (29.9%) followed by nurse assistants (22.9%) and pharmaceutical personnel (18.6%) (Table 1). Most of the study participants (63.5%) were having experience of more than 3 years of working as pharmaceutical outlet medicine dispensers and only 7.6% of the dispensers had experience of 0–1 year.

**Table 1:** Frequency and percentage distribution of the studied facilities and professional qualifications of the study participants (n = 422)

Variable	Count	Frequency (%)
Type of facility		
Dispensers from pharmacies	185	43.8
Dispensers from ADDO’s	237	56.2
Professional qualification		
Pharmacist	26	6.2
Pharmaceutical technician	28	6.7
Pharmaceutical assistant	24	5.7
ADDO trained	126	29.9
Nurse officer	92	21.8
Nurse assistant	97	22.90
Others	29	6.90

### Availability of sulfonamide based anti-malarial medicines

Sulfonamide based anti-malarial medicines were available in 76% of the community medicine outlets in Dar-es-salaam. The available sulfonamide based anti-malarial medicines include several products containing Sulfadoxine–pyrimethamine (Oroda and Fansidar^®^) and Sulfamethoxypyrazine–Pyrimethamine (Ekelfin, Malafin and Metakelfin^®^).

### Dispensers’ knowledge on SPs

**Fig. 1:**
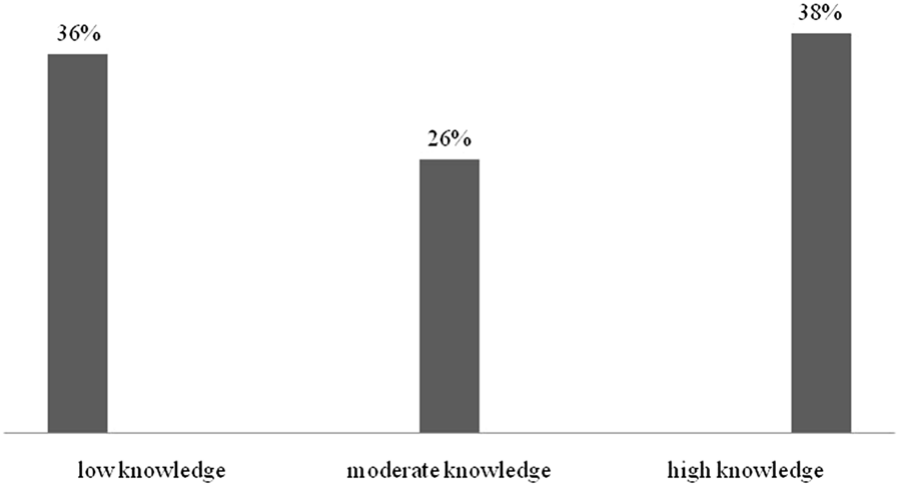
Percentage distribution of knowledge level regarding proper use of SPs among dispensers (n = 422)

This study revealed that majority of the pharmaceutical outlet dispensers 66.8% were aware that artemisinin-based combination therapy (ACT) is the first-line treatment of malaria. However, a significant number of dispensers 27% reported that SP is the recommended first line treatment of malaria. Of the study participants interviewed 90.5% were knowledgeable about some necessary advices to the patients dispensed with SP including the need to take a plenty of water to prevent kidney problems. Additionally, majority of the dispensers 55% were aware that the pregnant women are supposed to take at least 3 doses of SP for IPTp during the course of pregnancy. About 80% of the dispensers were knowledgeable that SP is reserved for IPTp. In general significant number of dispensers (36%) had poor knowledge about SPs and their current indications. The rest of the dispensers had moderate to high knowledge 270 (64%) (Fig. 1).

The study found that there was a statistically significant association between the level of knowledge of dispensers and the type of health facility from which the dispenser works (p = 0.004), working experience (p = 0.032) and the professional qualification (p = 0.001) as indicated in Table 2. The trend indicated that, the more the years of working the higher the knowledge and pharmacists were generally more knowledgeable than the rest of the professions. Dispensers from ADDO shops had relatively low levels of knowledge compared to pharmacy dispensers.

**Table 2:** Association of the knowledge regarding SPs and their indications with the selected demographic variables of retail pharmaceutical outlets dispensers (n = 422)

Knowledge count (%)					p-value
	Low level	Moderate level	High level	Number of dispensers	
Type of health facility					
Pharmacy	54 (29.9%)	45 (24.3%)	86 (46%)	185	0.004
ADDO shops	98 (41.3%)	65 (27.4%)	74 (31.2%)	237	
Working experience					
1 year	19 (59%)	6 (18%)	7 (22%)	32	0.032
2 years	50 (40%)	32 (26%)	40 (33%)	122	
3 years and above	83 (31%)	72 (27%)	113 (42%)	268	
Professional qualification					
Pharmacist	7 (27%)	3 (12%)	16 (61%)	26	0.001
Pharmaceutical technician	12 (43%)	10 (36%)	6 (21%)	28	
Pharmaceutical assistant	9 (38%)	5 (21%)	10 (42%)	24	
ADDO trained	47 (37%)	28 (22%)	51 (40%)	126	
Nurse officer	21 (23%)	25 (27%)	46 (50%)	92	
Nurse assistant	38 (39%)	32 (33%)	27 (28%)	97	
Others	18 (62%)	7 (24%)	4 (14%)	29	

### Dispensers’ actual practice

**Fig. 2:**
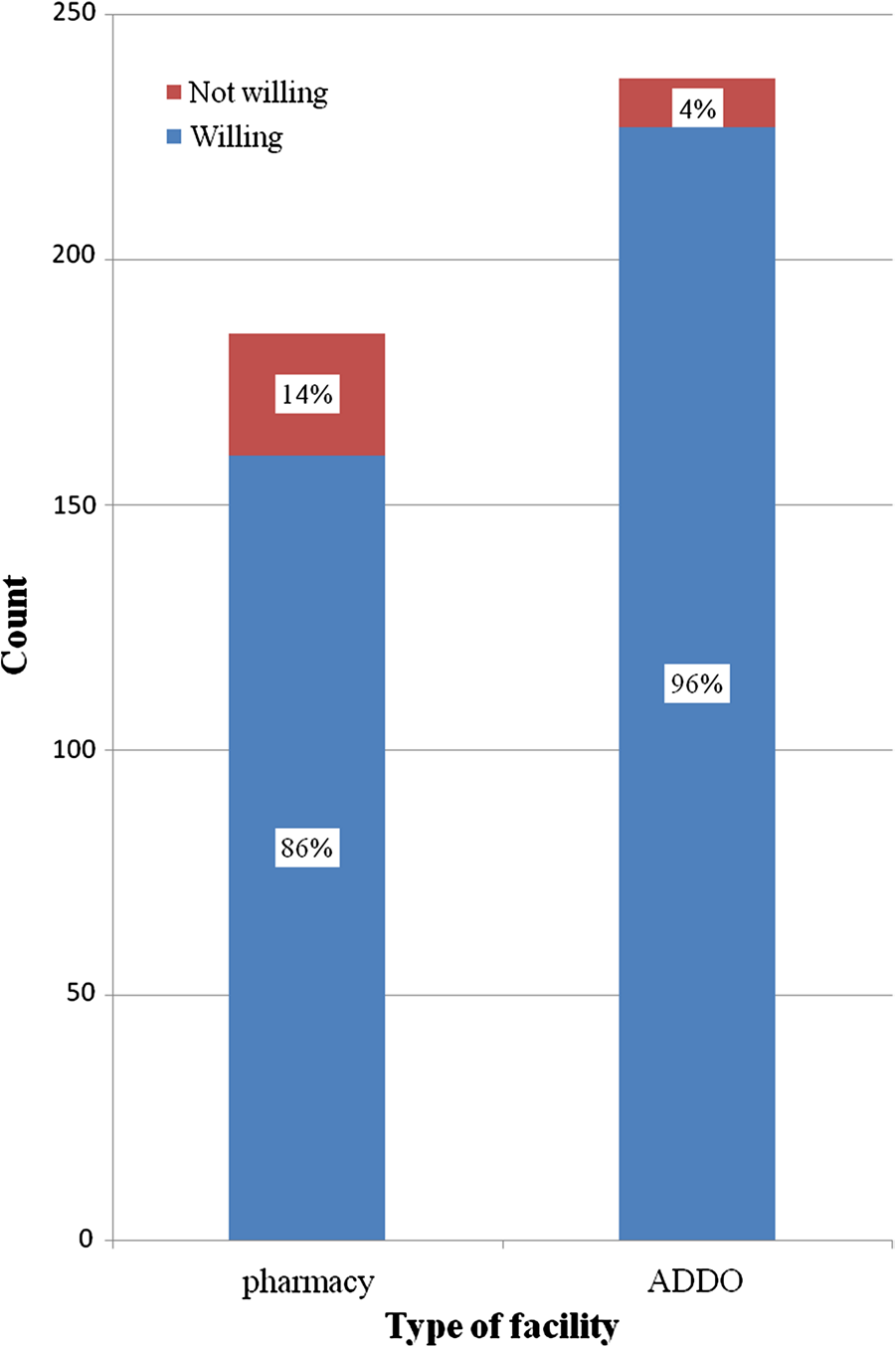
Dispensers’ willingness to dispense Sulfonamide bases anti-malarial medicines for treatment of malaria versus type of facility

On assessing the actual practice of the dispensers at the community medicine outlets the study revealed that 388 (92%) of the dispensers were ready to dispense SPs for the purpose of treatment of malaria. It was as low as only 4% and 14% of the ADDO shop and pharmacy dispensers respectively, were not willing to dispense sulfonamide-based anti-malarial medicines for treatment of malaria. There was a statistically significant association between practice and the type of facility whereby those who work in community pharmacies had relatively better practice than those working in ADDO shops (p-value = 0.001) (Fig. 2).

## Discussion

This study was conducted more than 10 years since SPs were initially reserved for IPTp SP in Tanzania. The study aimed to investigate the knowledge and actual practice of pharmaceutical outlet dispensers regarding SPs and their current indications. The majority of pharmaceutical outlet dispensers were found to have adequate knowledge about SPs and their indication but surprisingly almost all dispensers (92%) were ready to dispense the medicines for malaria treatment contrary to the available Tanzania malaria treatment guideline. These findings are comparable to the findings from a study which was conducted in Kenya by Rusk and his colleagues which revealed that most medicine retailers could identify the recommended first-line treatment for uncomplicated malaria but did not always ensure it or dispense to customers [27]. Another study conducted in Ghana to assess the knowledge of medicine outlets’ staff and their practices for prevention and management of malaria in Ghana and revealed less than 40% had adequate knowledge about the latest initiatives for malarial control, and only 21% could manage malaria cases as recommended by national guidelines [28].

Riley et al. demonstrated that retail pharmaceutical outlet dispensers sold SPs as treatment to only 11% of simulated clients who asked for treatment due to signs and symptoms consistent with malaria in Kenya [30]. Kioko et al. reported SPs to be relatively cheaper than ACT medicines and, therefore, likely to be misused for malaria treatment while some other studies have linked this poor dispensers’ practice with the pressure from the business owners and the clients [31]. Given the power that patient demand has on dispenser’s performance, future interventions focusing on the public education may positively influence dispensing of anti-malarials.

The effectiveness of IPTp using SP has been reported to be compromised in some countries [17]. The efficacy of SPs to clear peripheral parasites and prevent new infections during pregnancy is compromised in areas with > 90% prevalence of *Pfdhps*-K540E. Fortunately, at least three doses of IPTp using SP in these areas is still associated with increases in birth weight and maternal haemoglobin [16]. However, if misuse of SPs continues, IPTp SP may end up failing completely. Currently, there is no drug or drug combination that can substitute SP for IPTp, it is therefore important for the stake holders and the community to work hand in hand in promoting rational medicine use in order to preserve the available SP effectiveness.

In Tanzania, SPs are recommended only for IPTp and are generally delivered as part of the antenatal clinic package [32]. Wholesale and retail pharmaceutical outlets might stock SP for sale to private health facilities that provide antenatal care services or for women to purchase in the event of SP stock outs at antenatal clinics. Now, given that IPTp using SP is supposed to be provided as direct observed therapy (DOT) at the antenatal clinics [13], the researchers were also eager to see if these medications are still available at the retail pharmaceutical outlets. This research revealed that 76% of the retail medicine outlets still stock SPs. Kioko et al. reported the availability of SPs in rural western Kenya a region with moderate to high malaria to be 75% which is similar to findings from this study. Furthermore, they linked the high availability of SPs to selling to customers for treatment of uncomplicated malaria [31]. SP stock outs have been reported in Tanzania [33] and this could also have contributes to the observed high availability of SPs in the retail pharmaceutical outlets. A systematic review which was done by Thiam et al. came up with a number of barriers hampering implementation of IPTp policies including long waiting time at antenatal clinics, long distances to health facilities, poor service provider/client relations, and drug stock-outs [34]. To overcome these barriers, for some clients it will be easier for them to go to nearby retail pharmaceutical outlet to seek for these medications rather than going to the antenatal clinics and, therefore, this might be justified the observed availability of SPs in this study.

### Limitations of the study

With regard to the assessment of the actual practice of the dispensers, this study relied only on determining whether the dispenser was ready or not ready to issue SPs for the treatment of malaria. Worrying to be recognized as researchers during data collection using simulated client approach the researchers could not obtain some important dispensers’ demographic characteristics for detailed analysis.

## Conclusion

The study revealed that SPs were highly available in the retail pharmaceutical outlets. Majority of the medicine dispensers in the community pharmaceutical outlets were knowledgeable about SPs and their current indications but the percentage with low knowledge was still significantly high. Disappointingly, almost all dispensers irrespective of their levels of knowledge were willing to dispense SPs for treatment of malaria which is contrary to the available malaria treatment guideline.

Interventions focusing on the public education may positively influence proper dispensing of these anti-malarial medicines. Since majority of the dispensers were knowledgeable and yet were willing to dispense SPs for malaria treatment it is important to explore the motivations and drivers of dispensers to act contrary to their knowledge.
